# Differences in discontinuation of statin treatment in women and men with advanced cancer disease

**DOI:** 10.1186/s13293-018-0207-5

**Published:** 2018-10-20

**Authors:** Helena Bergström, Elsa Brånvall, Maria Helde-Frankling, Linda Björkhem-Bergman

**Affiliations:** 1 Department of Neurobiology, Care Sciences and Society (NVS), Division of Clinical Geriatrics, Karolinska Institutet, Blickagången 16, Neo floor 7, SE-141 83 Huddinge, Sweden; 2 Palliative Home Care and Hospice Ward, ASIH Stockholm Södra, Bergtallsvägen 12, SE-125 59 Älvsjö, Sweden; 3 Division of Clinical Epidemiology, Department of Medicine Solna, Karolinska Institutet, SE-171 77 Stockholm, Sweden

**Keywords:** Statins, Palliative care, Gender, Vitamin D, Cholesterol

## Abstract

**Background:**

Statins are often discontinued in patients with advanced cancer since the net effect of treatment is considered negative. However, guidelines concerning discontinuation of statin treatment are lacking. The aim of this study was to investigate any differences in time of discontinuation of statin treatment between men and women with advanced cancer disease.

**Methods:**

Medical records from 195 deceased palliative cancer patients from a previous study cohort were reviewed. Patients treated with statins 2 years before death were identified as “statin users.” The time of discontinuation of statin therapy was identified and correlated to time of death. Only patients that had incurable cancer disease at time of statin discontinuation were included in the analysis.

**Results:**

Fifty-four patients were identified as statin users, 29 women and 25 men. The average time span between discontinuation of statin treatment and time of death was significantly longer in women than in men, 10 months compared to 4 months (*p* < 0.01), with a range of 1–24 months among women and 1–12 months for men. All patients died due to their cancer disease. More men than women had a history of stroke or cardiac infarction (*p* = 0.02). There were no differences in age, socioeconomic factors, or survival time from study inclusion between men and women. There was no difference in self-assessed quality of life (QoL) between statin users who had discontinued statin treatment and those who are still on treatment. Men generally assessed their QoL lower than women in this study (*p* = 0.03).

**Conclusion:**

Statin treatment was discontinued earlier in women than in men in patients with advanced cancer. The data suggest that statins may be discontinued earlier in men as well, since earlier discontinuation did not affect cardiovascular mortality.

## Background

In palliative care, the overall aims are prevention from and relief of suffering in patients with life-threatening illnesses, as well as improvement of quality of life. The goal is neither to prolong life nor to hasten death. Therefore, it is important that side effects of any medical treatment do not outweigh possible beneficial effects.

Statins (3-hydroxy-3-methylglutaryl-coenzyme A reductase inhibitors) are efficient in lowering cholesterol in the circulation, and large clinical trials have shown that statin treatment leads to prolonged survival in risk groups due to reduced cardiovascular disease burden [1]. However, since the preventive effects of statins on cardiovascular disease require long-term treatment, beneficial effects of statins are not to be expected in terminally ill cancer patients. Statin treatment is therefore often discontinued in patients with advanced cancer disease. However, there are no clear guidelines for statin discontinuation in palliative cancer patients. Since guidelines are lacking, the decision regarding discontinuation often depends on how the individual physician values risk and benefit, as well as on local practices. Interestingly, during recent years, several epidemiological studies have suggested a possible cancer-preventive effect of statin treatment, as well as a reduced cancer-related mortality [2–7].

Statins are generally considered to be well-tolerated drugs. However, during recent years, several reports have demonstrated that statin-induced muscular side effects, such as myalgia, are more common than first predicted in clinical trials [8]. In fact, 10–15% of patients prescribed with statins may experience adverse muscle symptoms [9, 10], and the risk of myalgia is more common in women than in men [11]. One study carried out in cancer patients showed that in patients > 80 years of age, statin use was associated with a significant increase in self-reported pain [12].

Low vitamin D levels (25-hydroxyvitamin D) have been linked to increased risk of statin-induced myopathy in several observational studies [13–15]. In a Swedish study, we could demonstrate that 25-hydroxyvitamin D levels < 50 nmol/L were associated with a fourfold increase in risk of statin-induced myopathy [14]. In addition, we and others have shown that palliative cancer patients generally have low 25-hydroxyvitamin D levels [16–20]. Thus, palliative cancer patients have an increased risk of statin-induced myopathy.

The aim of this study was to investigate whether there was a difference in time of statin discontinuation before time of death between women and men admitted to a palliative home care unit in Sweden. We also reviewed the literature regarding discontinuation of statins in terminally ill cancer patients.

## Methods

### Study cohort

We collected data from 195 deceased cancer patients who had participated in two studies on vitamin D status at our Palliative Care Unit in Stockholm, Sweden (ASIH Stockholm Södra), between 2014 and 2016. This Palliative Care Unit includes both advanced medical home care, i.e., patients receiving medical care in their own homes, and a hospice ward for in-patients. Results from these studies have been published elsewhere [16, 19]. Both studies included patients suffering from advanced, incurable cancer disease, who were admitted to our palliative unit, had a lifetime expectancy of more than 1 month, and who were able to assess quality of life (QoL) with the Edmonton Symptom Assessment System (ESAS) [21]. ESAS is a scale from 0 to 10 where 0 is the best QoL and 10 is the worst QoL.

The first study, carried out in 2014–2015, was an observational study where 25-hydroxyvitamin D was measured and related to opioid dose, infectious burden, and QoL [16]. In the second study, carried out in 2015–2016, the same parameters were studied in a cohort where patients with 25-hydroxyvitamin D < 75 nmol/L were offered treatment with vitamin D 4000 IE/day for 3 months [19]. Patients from both of these studies were merged into one combined cohort in which we have data on age, sex, cancer disease, survival time from inclusion, QoL, opioid dose, antibiotic consumption the month before and after inclusion, and levels of albumin, C reactive protein (CRP), and 25-hydroxyvitamin D at time of inclusion. The patients were recruited consecutively and could have any type of cancer.

From this combined cohort, we identified all 195 patients who are now deceased. Due to of the joint medical records in Stockholm County health care system, all prescribed drugs during the last 10 years can be identified. In the cohort of 195 patients, we identified 55 patients who had been prescribed statin treatment for more than 1 month during the last 10 years. However, one patient had discontinued statin use before being diagnosed with cancer and was not included in the analysis. Fifty-four patients discontinued statin treatment at some point in time after being diagnosed with incurable cancer disease and were included in the analysis. The earliest discontinuation of statin treatment in relation to death was 24 months before death.

### Data extraction

Data from medical records were collected regarding statin type and dose, indication for statin treatment (primary or secondary prevention), cardiovascular morbidity, time for discontinuation of statin treatment, reason for statin discontinuation if stated, responsible physician, time of death, and the major cause of death according to the death certificate. In addition, we collected data on place of residence and level of education/profession if stated in the medical record. Data regarding age, sex, cancer diagnosis, QoL, and levels of 25-hydroxyvitamin D, CRP, and albumin had been extracted previously.

### Statistical analysis

Statistical analyses were performed using Graph Pad Prism vs 6.0. Comparisons of termination of statin treatment in relation to death; age; levels of CRP, albumin, and 25-hydroxyvitamin D; survival time; and QoL were performed using Student’s *t* test. The data showed Gaussian distribution. Comparisons of proportions of women and men treated with statins due to secondary or primary prevention were performed using Fisher’s exact test.

### Ethics statement

The original studies were approved by the local Ethical Committee at Karolinska Institutet, Stockholm, Sweden (Dnr: 2014/455-31/4 and 2015/776-31), and were performed in accordance with the Declaration of Helsinki. Written informed consent was obtained from all patients before inclusion in the original studies, including permission of review of medical records.

## Results

### Study group of statin users

Out of the 54 statin users, there were 29 women and 25 men. Baseline characteristics, including age, laboratory data, type of statin, indication for statin use, and presence of previous cardiovascular event are presented in Table 1. Distribution of types of cancers is presented in Table 2. There were no differences in age and levels of albumin, CRP, or 25-hydroxyvitamin D between the men and women (Table 1). Simvastatin was the most common statin used in both men and women and is still the most frequently used statin in Sweden. At time of inclusion in the original vitamin D studies, only three women (10%) and seven men (28%) were still on statin treatment. One woman and four men were after inclusion in the study supplemented with vitamin D 4000 IE/day for up to 3 months.

**Table 1:** Demographic data of the 54 statin users in the study cohort. Age, lab values, and survival time is at time of inclusion in the original studies [16, 19]. All values are presented as mean ± SD if not specified otherwise. *P* values show statistically significant differences between women and men. Survival time is from time of inclusion in the original vitamin D study

	Total (*N =* 54)	Men (*N =* 25)	Women (*N =* 29)	*p*
Patients’ characteristics				
Age (years)	72 ± 10	71 ± 11	73 ± 8	ns
Age > 75 years, *n* (%)	20	10	10	ns
Albumin (g/L)	27 ± 7	27 ± 6	27 ± 7	ns
CRP (mg/L)	65 ± 27	63 ± 126	66 ± 82	ns
25-OHD (nmol/L)	45 ± 29	43 ± 26	47 ± 32	ns
ESAS QoL	5.7 ± 2.5	6.5 ± 2.4	5.0 ± 2.4	0.03
History of stroke, *n*	6	4	2	ns
History of myocardial infarction, *n*	12	8	4	ns
Survival time (months)	3.3 ± 4.5	2.2 ± 2.5	4.3 ± 5.5	ns (0.10)
Highest educational level				
University degree	4	2	2	ns
No university degree	30	14	16	ns
Unknown	20	9	11	ns
Indication for statin use				
Primary prevention, *n*	36	13	23	
Secondary prevention, *n*	18	12	6	0.03
Type of statin, *n* mean dose (mg)				
Simvastatin	41 28 mg	21 31 mg	20 24 mg	ns
Atorvastatin	10 23 mg	3 27 mg	7 19 mg	ns
Rosuvastatin	2 10 mg	0	2 10 mg	ns
Pravastatin	1 20 mg	1 20 mg	0	ns

*ESAS QoL*: The Edmonton Symptom Assessment System, Quality of life assessment, a scale from 0 to 10

*CRP*: C reactive protein, *25-OHD*: 25-hydroxyvitamin D, *ns*: non-significant

**Table 2:** Types of cancers in the 54 statin users in the study cohort. Values show number of patients

Type of cancer	All (*N =* 54)	Men (*N =* 25)	Women (*N =* 29)
Lung	13	6	7
Breast	5	1	4
Gastrointestinal	12	7	5
Pancreas, liver	7	1	6
Sarcoma	1	0	1
Urothelial	5	5	0
Hematological	3	3	0
Brain tumor	2	2	0
Gynecological	3	0	3
Melanoma	1	1	0
Ear-nose and throat	2	0	2

All patients were living in the southern part of Stockholm in low- to middle-income areas. There was no difference between men and women in place of residence. The educational level was also similar between men and women. Only 7% (*n* = 2) of the men and 7% (*n* = 2) of the women had a university degree.

There was no statistically significant difference in survival time between women and men after inclusion in the vitamin D studies (Table 1).

### Statin discontinuation

**Fig. 1:**
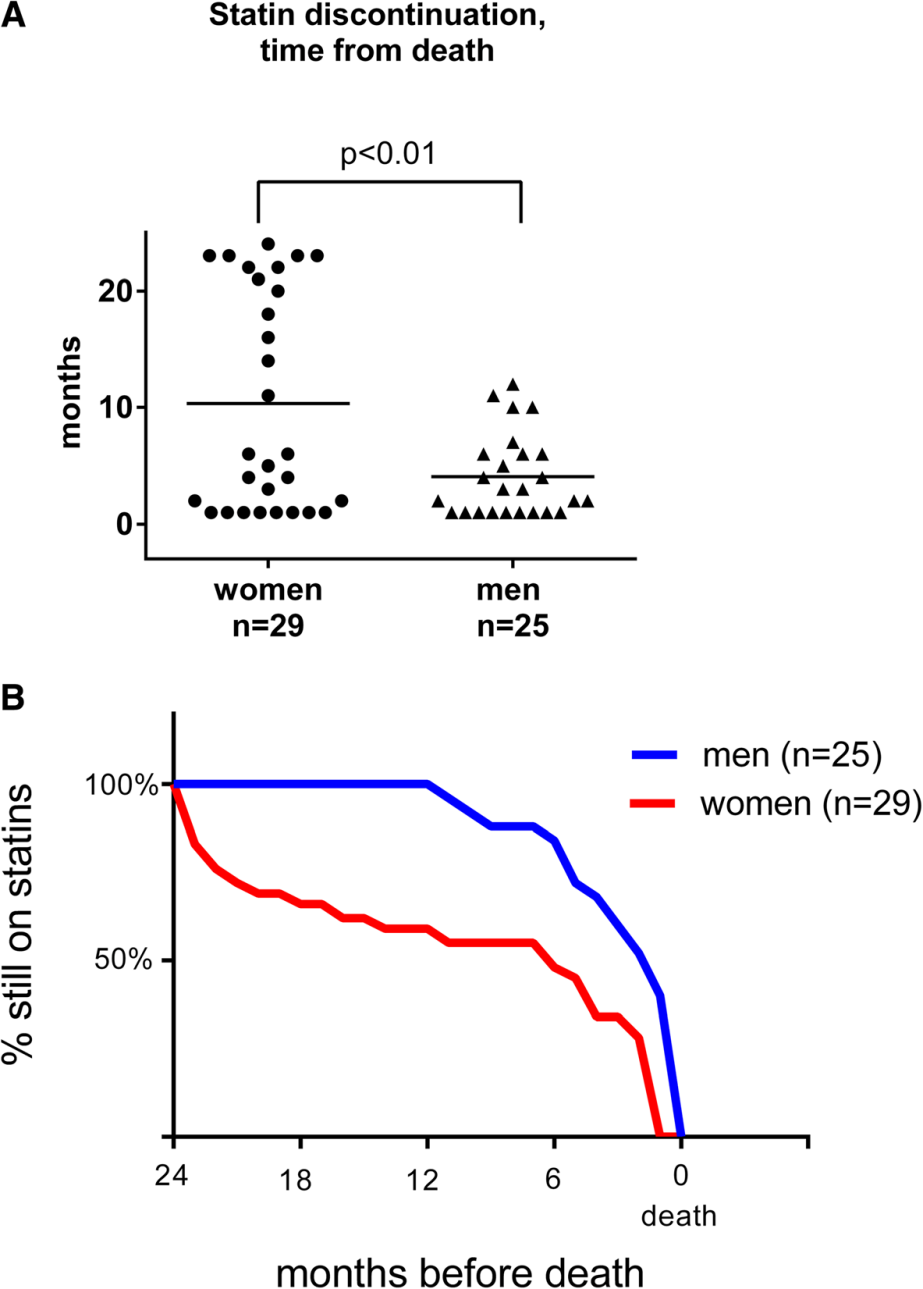
Time for statin discontinuation in relation to death in 29 women (dots) and 25 men (triangles) with advanced cancer, admitted to a palliative care unit. **a** Statins were on average terminated earlier in women than in men, statistical analysis using Student’s *t* test showed *p* < 0.01. Time-point “1 month” means 1 month or less before death. **b** Time-graph over when statin was discontinued in men and women in relation to death

**Fig. 2:**
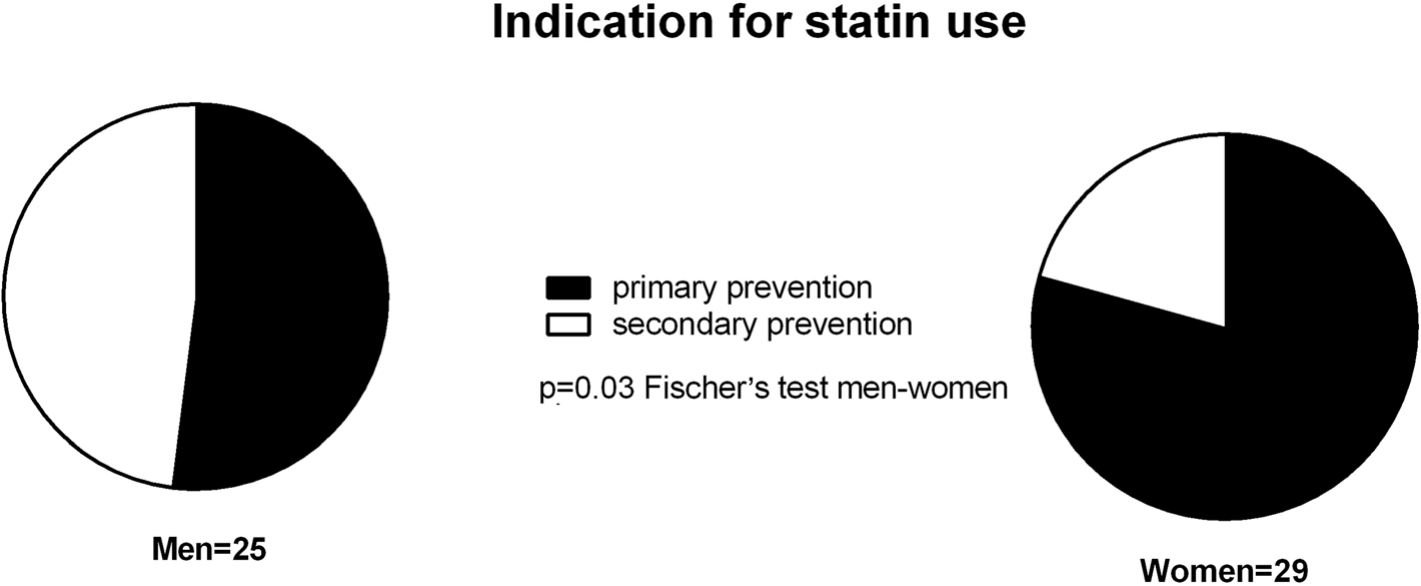
Indication for statin treatment in 29 women and 25 men with advanced cancer admitted to a palliative care unit. The indication for statin treatment was secondary prevention after a cardiovascular event in 6 of 29 women (21%) and in 12 of 25 men (48%), a difference that was statistically significant (*p* = 0.03)

The average time from statin discontinuation to death was significantly longer in women than in men; average 10 ± 9.4 months compared to 4 ± 3.5 months (*p* < 0.01) (Fig. 1a). As shown in Fig. 1b, half of the women had their statin discontinued 24–18 months before death, while no men had their statin discontinued earlier than 12 months before death. Indication for statin treatment was secondary prevention after a cardiovascular event in 6 out of 29 women (21%) and in 12 out of 25 men (48%) (Fig. 2), a difference that was statistically significant (Fisher’s exact test *p* = 0.03).

It should be noted that 13 men and 23 women received statins as primary prevention, although they were suffering from a life-limiting disease (Table 1). There was no trend towards earlier discontinuation of statin treatment where the indication was primary prevention. In fact, seven of the nine men and seven of the eight women who continued their statin treatment until 1 month before death were prescribed statins as primary prevention.

**Fig. 3:**
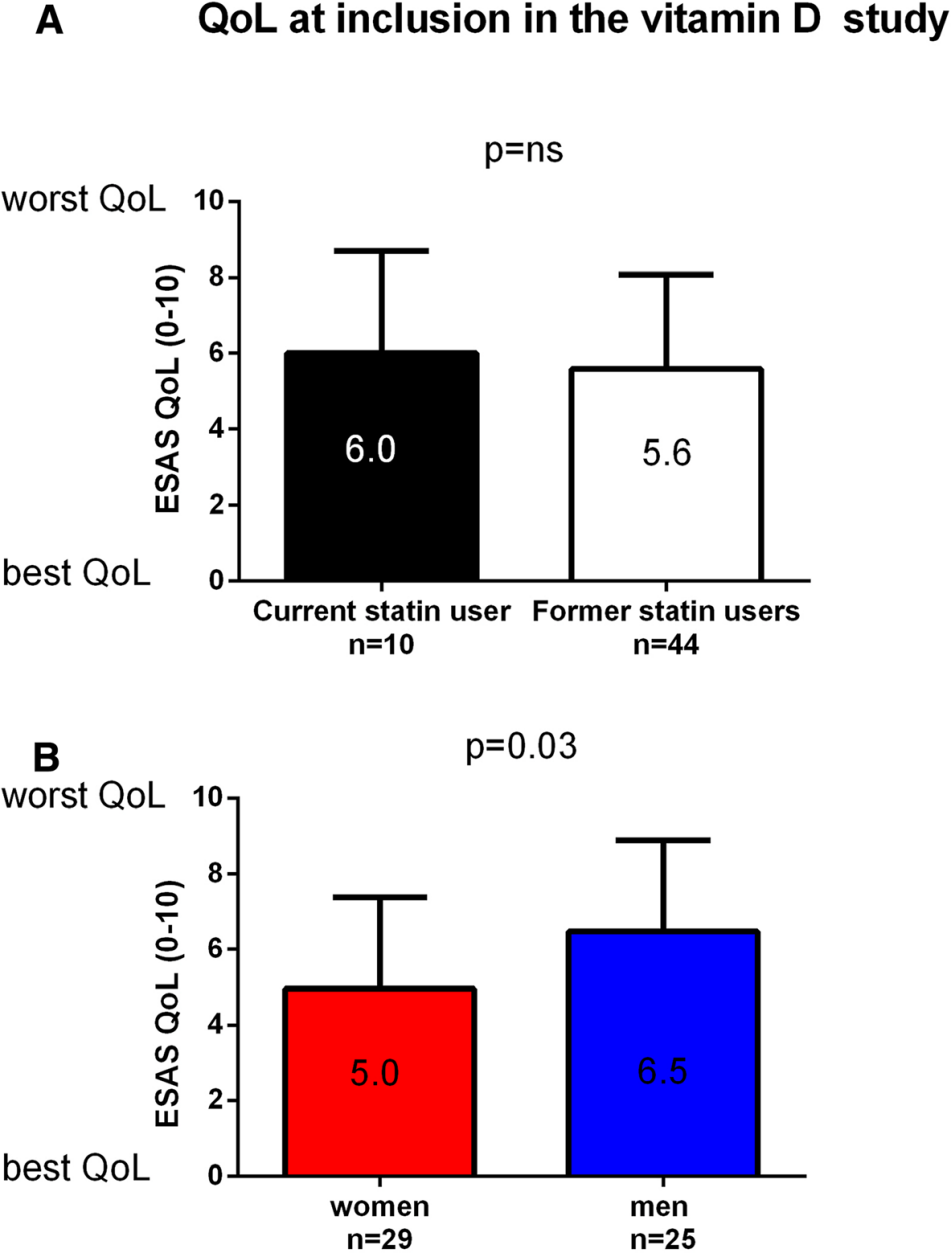
Average assessed quality of life (QoL) in **a** current (*n* = 10) and former (*n* = 44) statin users with advanced cancer admitted to Palliative Care. **b** Men (current and former statin users *n* = 25) generally assessed their QoL at inclusion in the study worse than women (current and former statin users *n* = 29), Student’s *t* test *p* = 0.03. QoL was measured using the Edmonton Symptom Assessment System (ESAS), a scale from 0 to 10 where 0 is the best QoL and 10 is the worst QoL

At time of study inclusion, 10% of the women and 28% of the men were still on statin treatment. There was no difference in self-assessed QoL in the patients with ongoing statin treatment and those who had terminated statin treatment (Fig. 3a). However, men generally assessed their quality of life lower than women, with ESAS differing 1.5 points between the groups (*p* = 0.03) (Fig. 3b). In 48 of the 54 cases, a physician at the Palliative Unit had discontinued statin treatment. In six cases, a physician at the hospital (oncologists and geriatricians) had terminated the treatment. In summary, statin was discontinued by 29 different physicians among the 54 patients.

### Reasons for statin discontinuation

Medical records did not state statin-induced myopathy or other adverse reactions as the cause of statin discontinuation in any of the patients. In 16 women and 12 men, there was no notification in the medical record about reason for statin discontinuation. In 26 patients, there was some notion in the patients’ medical records as to why statin use was discontinued (Table 3).

**Table 3:** Causes of statin termination as stated in the medical records of the 54 statin users in the study cohort. Values show number of patients and % within parenthesis

Reasons for termination of statin treatment	Total (*n =* 54) *n* (%)	Men (*n =* 25) *n* (%)	Women (*n =* 29) *n* (%)
Risk for harm greater than benefit	11 (20)	8 (32)	3 (10)
Short lifetime expectancy	10 (19)	4 (16)	6 (21)
Cachexia	3 (6)	1 (4)	2 (7)
Fatigue	1 (2)	0 (0)	1 (3)
Dysphagia	1 (2)	0 (0)	1 (3)
Not specified	28 (52)	12 (48)	16 (55)

The most common causes in both men and women were “risk for harm greater than benefit” (20%) or “short life-time expectancy” (19%).

### Cause of death

All patients died due to their cancer disease according to the death certificate, and no one died due to cardiovascular events.

## Discussion

In this study, statin treatment was generally terminated earlier in women with advanced cancer as compared to men. This difference was not explained by age, sociodemographic factors, cancer type, or expected survival time. One possible reason for continued statin treatment in men could be a difference in indication for statin use, since secondary prevention was more common in men. Physicians are likely to be more prone to continue statin treatment in a patient with an earlier cardiovascular event where the risk reduction with statin is expected to be more pronounced. However, the majority of both men and women who were on statin treatment until the last month of life had statin treatment as the primary prevention, indicating that previous cardiovascular events were not part of the assessment when statins should be discontinued. This is in line with the findings in an earlier retrospective study (*n* = 539) in poor-prognosis cancer patients showing that statin treatment was continued despite life-limiting disease even when the indication was primary prevention [22].

Half of the women had their statin discontinued 24–18 months before death, while no men had their statin discontinued earlier than 12 months before death. It could be hypothesized that the life expectancy was overestimated in the male patients or underestimated in the women. Another possible explanation is that men had their cancers evaluated as incurable at a later stage than the women did in the cohort.

Interestingly, men generally assessed their QoL lower than women, and there was no difference in self-assessed QoL between former and current statin users, i.e., statin use was not associated with increased QoL. No patient died from a cardiovascular event. Thus, there was no apparent benefit for the men in this study to continue with statin treatment.

To our knowledge, no previous study has been carried out to study gender differences in statin discontinuation in patients with advanced cancer disease. In studies on statin discontinuation in non-cancer patients, the majority of the participants have been men [23].

In a study by Kutner and co-workers, 381 statin users with life-threatening illness were randomized to discontinue statins (*n* = 189) or to continue statin use (*n* = 192). In this study, statin discontinuation resulted in increased QoL and did not affect survival time [24]. The same research team also carried out a study on patient perceptions of discontinuation of statin therapy (*n* = 297) [25]. This study showed that patients with life-threatening illnesses were mostly positive to discontinue statin-treatment, including spending less money on medications and having a better quality of life. Fewer than 5% of the participants expressed concerns about the discontinuation, including feelings of being abandoned by their physician [25].

There are well-established differences between the sexes in the prevalence of risk factors for cardiovascular disease, such as hypertension, hypercholesterolemia, and diabetes. Also, women are more likely to be older and have more co-morbidities than men at the time of onset of cardiovascular disease [26]. In addition, there are sex differences in sociodemographic factors such as educational level and disposable income, and it has been shown that such factors influence the likelihood of continuing statin treatment or not [27]. However, in the current cohort, there were no significant sociodemographic differences between men and women when measured as educational level and place of residence.

The patterns of statin treatment differ between the sexes, as women are less likely to be both prescribed [28] and dispensed statins for secondary prevention [29]. In addition, women report a higher frequency of adverse drug reactions in general as compared to men [30]. In a prospective observational study of statin-induced myopathy in an outpatient setting, women reported a higher frequency of myopathy and other adverse drug reactions, as well as a more negative impact of daily life activities [11]. Combined, these factors may contribute to a lower likelihood of adequate treatment with statins for women and may also contribute to a higher propensity for statin discontinuation among women in our cohort.

It has been suggested in epidemiological studies that statin use may be associated with prolonged survival in several cancer types, including prostate, colorectal, endometrial, liver cancer, and myeloma [2–7]. In vitro and animal experiments have also shown that statins may inhibit progression of cancer cells [31–33]. However, population-based observational studies may be associated with methodological biases and there are so far no large, interventional studies showing prolonged survival in cancer patients taking statins, nor any general recommendations to continue statin treatment for cancer-preventive effects. Also importantly, such an inhibiting effect on cancer progression is hypothesized to be long-term and thus not likely to be of any major importance in a late palliative setting.

A disadvantage of continued statin therapy, besides the general risk of myopathy [9, 10] and increased pain [12], is the risk of drug-drug interactions. All lipid-soluble statins, e.g., simvastatin and atorvastatin, are metabolized by hepatic cytochrome P450 (CYP) enzymes, while the water-soluble pravastatin is mainly eliminated in the urine. In cancer patients with concomitant drugs that inhibit CYP3A4, e.g., fluconazole, there is a risk of drug-drug interactions with increased concentrations of lipid-soluble statins and subsequently increased risk of adverse events and myotoxicity [34, 35].

There are several limitations to this study. First, the sample size is small and the data was collected at one single center. Thus, the findings presented here need to be confirmed in a larger and preferably multicenter study. Second, we have incomplete data on socioeconomic factors in men and women that might affect when statins are discontinued, and the patients’ opinions regarding statin discontinuation are not assessed. Furthermore, data on the history of smoking, diabetes, and other co-morbidities are lacking.

Despite the limitations, this is to our knowledge the first study in which sex differences in statin discontinuation is studied in patients with advanced cancer disease and the results from this pilot-study could be used for power calculation to optimize the design of a future study,.

## Conclusion

Statin treatment in patients with advanced cancer was discontinued earlier in women than in men. Discontinuation of statins did not affect cardiovascular mortality and did not decrease QoL suggesting that statins may be safely terminated earlier in men as well. However, these findings need to be confirmed in a larger study that is now in the planning phase at our Palliative Unit at ASIH Stockholm Södra.

## Abbreviations

25-OHD: 25-Hydroxyvitamin D
CRP: C reactive protein
ESAS: Edmonton Symptom Assessment System
ns: Non-significant
QoL: Quality of life
